# Should Post Kidney Transplantation Hyperlipidemia Considered a Risk Factor for Graft Function?

**Published:** 2010-08-01

**Authors:** G. Pourmand, A. Saraji, S. Dehgani, A. Mehrsai, M. Nikoobakht, M. Talibnajad, E. Razeghi, M. Rahbar, H. Hosseini, N. Pourmand, Sh. Pourmand, M. Zahedikia, M. Porhussein, F. Heidari

**Affiliations:** *Urology Research Center, Tehran University of Medical Sciences, Tehran, Iran*

**Keywords:** Hyperlipidemia, Post kidney transplantation, Grafts survival

## Abstract

Background: Hyperlipidemia is a common problem after kidney transplantation.

Objective: To uncover the real impact of post kidney transplantation hyperlipidemia on graft function and survival, and to determine whether it is just a biochemical phenomenon after using immunosuppressant or a part of disease pathology.

Methods: 330 kidney transplants were managed in Sina Hospital Kidney Transplantation Unit affiliated to Tehran University of Medical Sciences, Tehran, Iran from September 1994 till February 2010. The demographic characteristics of the patients, causes of chronic kidney diseases, history of pretransplantation dialysis, pretransplantation comorbidities (*e.g.*, hypertension, diabetes mellitus [DM], hyperlipidemia and coronary artery disease), rejection episodes, status of infection with cytomegalous virus [CMV], post-transplantation DM, hyperlipidemia, ischemic heart disease [IHD], and graft and patient survival were recorded. A serum creatinine level >2 mg/dL was considered as “graft deterioration,” and return to dialysis as “graft loss.” According to the presence or absence of post kidney transplantation hypercholesterolemia (>200 mg/dL) or hypertriglyceridemia (>200 mg/dL), the patients were classified into “hyperlipidemic” or “non-hyperlipidemic.” The presence of clinical or paraclinical coronary artery disease was also determined in both groups.

Results: The incidence of hyperlipidemia elevated from 8% to 50% before and after transplantation. 2.7% developed clinical IHD. 13% of hyperlipidemics and 22% of non-hyperlipidemics developed graft deterioration. Among hyperlipidemics with deteriorated grafts 40% had premorbid diseases, 68% had CMV infection and 82% had hypertension. Only 22% had previous acute rejection and 27% received deceased kidney transplant.

Conclusions: post kidney transplantation hyperlipidemia is just an associated phenomenon secondary to the use of immunosuppressant medications, which have no obvious impact on renal graft function and can be easily controlled by instituting dietary modifications and use of modern antilipid medications. Post kidney transplantation CMV infection and hypertension are considered as the main threatening risk for renal graft—even more dangerous than acute or chronic rejections.

## INTRODUCTION

Innovative developments in immunosuppressant regimens have markedly improved patient and graft survival at one year. However, after the first year of transplantation, cardiovascular morbidity and mortality are the major causes of death with functioning graft. Among middle aged patients with a functioning graft, mortality rate due to coronary artery diseases is approximately 0.6% per year [1], which is more than five times the value in the general population aged 45–64 years [2]. More than half of the recipients death after the first year of renal transplants in North America and Europe are due to cardiovascular causes [3-5]. Premature death is the most common cause of mortality in patients with a functioning graft [6] mostly due to accelerated atherosclerosis [7]. Several factors lead to new changes in lipid abnormalities in post-transplantation period. Dyslipidemia occurred at the time of development of chronic kidney diseases [8] is a risk factor. Dialysis therapy results in vasculopathy which is directly proportional to the duration of dialysis [9-11]. Other risk factors include increased incidence of hypertension in post-transplantation period [12-14], preexisting cardiovascular and diabetic diseases at the time of transplantation [15, 16], new onset post-transplantation diabetes mellitus (DM) and insulin resistance syndrome [17,18], athrogenic effect of corticosteroids and calcineurins inhibitors [19,20].

Improved survival in the general population following treatment with lipid lowering agents has been attributed to their pharmacological effects to lower the cholesterol level [21,22]. Whether hyperlipidemia *per se* adversely affect the patient and graft survival in recipients of organ transplants remains a matter of debate [7,23,24]. Early studies failed to demonstrate any significant association between the lipid levels and cardiovascular morbidity and mortality rates [25]; no association was also found between post-transplant hyperlipidemia and patient or graft survival [23,26]. We conducted this study to determine the impact of lipid control on kidney graft survival, and whether strict lipid control by lipid lowering medications as a fixed protocol after kidney transplantation is mandatory.

## PATIENTS AND METHODS

This retrospective study reviewed medical records of 330 kidney transplantation patients managed by the same nephrology, urology, nursing and laboratory team in Sina Hospital Kidney Transplantation Unit affiliated to Tehran University of Medical Sciences, Tehran, Iran from September, 1994 to February, 2010. In addition to the demographic characteristics of the patients, we also assessed patients’ body mass index (BMI), cause of chronic kidney diseases, type and duration of dialysis, pretransplantation comorbidities (*e.g.*, hypertension, DM, hyperlipidemia and coronary artery disease), rejection episodes, post-transplantation immunosuppressant regimen, cytomegalovirus (CMV) infection, post-transplantation DM, hyperlipidemia, ischemic heart disease (IHD), and graft and patient survival. A serum creatinine level >2 mg/dL was considered “graft deterioration,” and return to dialysis as “graft loss.” According to the presence or absence of post-kidney transplantation hypercholesterolemia (>200 mg/dL) or hypertriglyceridemia (>200 mg/dL), the patients were classified into “hyperlipidemic” or “non-hyperlipidemic.” The presence of clinical or paraclinical coronary artery disease was also determined in both groups. Patients were followed monthly (1st year), every two months (2nd year) and every three months thereafter. CoX-2 statistical method was used for data analysis.

## RESULTS

Half of 330 patients (64% males, 36% females) did not develop hyperlipidemia. The remaining half (59% males, 41% females) developed hyperlipidemia after kidney transplantation, from whom 27 (8%) patients had pretransplantation hyperlipidemia. The mean age in non-hyperlipidemic and hyperlipidemic group was 37 (range: 9-64) and 41 (range: 9-63) years, respectively (Table 1). Of 330 studied patients, 9 (2.7%; six men and three women aged 24–63 years) developed clinical IHD, of whom one died of coronary vascular disease, two had deteriorated graft, five were hyperlipidemics and four were non-hyperlipidemics. Six percent of studied patients underwent living related transplantation, 14% of them received the transplant from cadaveric and 80% were from living unrelated donors (Table 1). Twenty-one percent of patients were on azathioprine premedication. This immunosuppressive regimen was switched to mycophenolate mofetile. On the other hand, 79% were on mycophenolate fometile from the beginning (Table 2). Thirty-seven percent of non-hyperlipidemics patients had premorbid conditions (DM, IHD, or hypertension) before transplantation; the prevalence for hyperlipidemic group was 46%. Forty-two (13%) patients were preemptively transplanted, while the remaining patients were on hemodialysis for 1–120 months. Only 3% were overweighted (BMI>24 kg/m^2^); all of them were hyperlipidemics. Among the post-transplantation hyperlipidemic patients, 11% were preemptively transplanted and 89% were on dialysis program; 46% of them had premorbid diseases, all of the pre-transplantation hyperlipidemics were put in this group; 24% had acute rejection in the early post-transplantation period; 38% were developed at least one attack of CMV infection; and 76% were hypertensive (Table 3). Eighty-one percent of transplants were donated by living unrelated, 4% by living related and 11% by deceased donation. Thirty-five percent of those who were taking azathioprine were hyperlipidemics.

**Table 1:** Demographic charecteristics and type of donation pre- and post-kidney transplantation in hyperlipidemic and non-hyperlipidemic groups

	**Post-transplant non-hyperlipidemia**	**Post-transplant hyperlipidemia**	**Graft deterioration or loss with non-hyperlipidemia**	**Graft deterioration or loss with hyperlipidemia**	**Mortality and morbidity due to IHD and hyperlipidemia**
Age	37yrs(9-64)	41yrs(9-63)	37yrs(18-60)	40 yrs (16-50)	46yrs(24-63)
Males	64%	59%	69%	64%	66%
Females	36%	41%	31%	36%	33%
Overweight	0/165	4/165(2.5%)			
Pre-transplant hyperlipidemia	Non	27/330			
Living related	8/165 (5%)	7/165 (4%)	1/37 (3%)	2/22 (4.5%)	0/9 (0%)
Living unrelated	146/165 (88%)	134/165 (81%)	34/37 (92%)	13/22 (68%)	9/9 (100%)
Cadaver	11/165 (7%)	18/165 (11%)	2/37 (5%)	7/22 (27%)	0/9 (0%)

**Table 2:** Distribution of hyperlipidemia before and after MMF era

	Hyperlipidemia	Non-hyperlipidemia	Hyperlipidemia control time	Age
AZA*	25/70(35%)	45/70(64%)	>24 m	35yrs
MMF†	110/260(42%)	150/260(57%)	<24m	36.5yrs

* MMF: mycophenolate mofetile,

† AZA: azathioprine

**Table 3:** Kidney graft deterioration risk factors in hyperlipidemic and non-hyperlipidemic groups

	**Post-transplant non-hyperlipidemia**	**Post-transplant hyperlipidemia**	**Graft deterioration or loss with non-hyperlipidemia**	**Graft deterioration or loss with hyperlipidemia**	**Mortality and morbidity due to IHD and hyperlipidemia**
Acute rejection episodes	25/165 (15%)	40/165 (24%)	9/37 (24%)	9/22 (40%)	2/9 (22%)
Premorbidity (DM, HTN, IHD)	60/165 (37%)	76/165 (46%)	16/37 (43%)	9/22 (40%)	8/9 (89%)
CMV infection	39/165 (24%)	63/165 (38%)	9/37 (24%)	15/22 (68%)	1/9 (11%)
Hypertension	36/165 (22%)	126/165 (76%)	9/37 (38%)	18/22 (82%)	6/9 (66%)
Normotensive	129/16 (78%)	39/165 (24%)	20/37 (54%)	4/22 (18%)	3/9 (33%)

Among non-hyperlipidemic patients, 14% were preemptively transplanted and 86% were on dialysis program (Table 4). Thirty-seven percent of them had premorbid conditions (none of the patients with pre-transplantation hyperlipidemics were enrolled in this group), 15% had acute rejection in early post-transplantation period, 24% developed at least one attack of CMV infection, 22% were hypertensives, 88% were donated by living unrelated, 5% by living related and 7% by deceased donation. Sixty-four percent of those who were taking azathioprine were non-hyperlipidemic.

**Table 4:** Pre-transplantation dialysis in hyperlipidemic and non-hyperlipidemic groups

**No**	**Pre-transplant hyperlipidemia**	**Post-transplant non hyperlipidemics**	**Post-transplant hyperlipidemia**	**Graft deterioration or loss with hyperlipidemia**	**Mortality and morbidity due to IHD and hyperlipidemia**
Total	27/330 (8%)	165/330 (50%)	165/330 (50%)	22/165 (13%)	9/330 (2.7%)
Preemptive transplantation	9/42 (21%)	23/165 (14%)	19/165 (11.5%)	7/22 (31%)	non
After dialysis transplantation	18/288 (6%)	142/165 (86%)	146/165 (88.5%)	15/22 (69%)	9/9(100%)

While 13% (n=22) hyperlipidemic patients developed graft deterioration, 22% (n=37) of non-hyperlipidemics did so (p>0.05); 40% of the deteriorated hyperlipidemic grafts were associated with premorbid conditions. Forty percent had evidence of previous acute rejection episodes, 68% complained at least one CMV infection who were admitted and treated perfectly (Fig 2). Eighty-two percent were hypertensive, 68% received transplants from living unrelated, 4.5% from living related and 27% from deceased donors (Fig 3).

**Figure 1:**
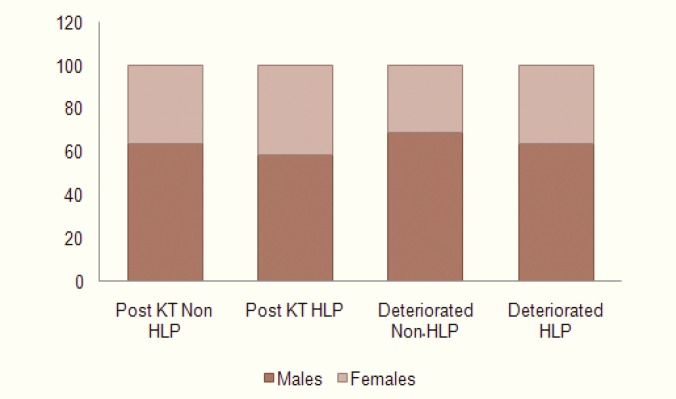
Sex distribution among hyperlipidemics (HLP) and Non-hyperlipidemics (Non-HLP) after kidney transplantation

**Figure 2:**
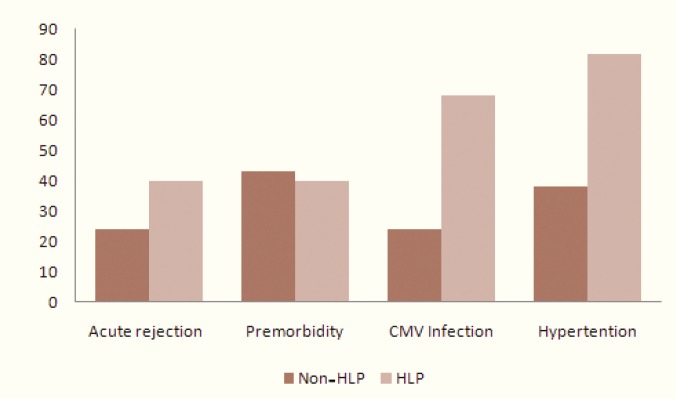
distribution of kidney graft deterioration risk factors in hyperlipidemics (HLP) and Non-hyperlipidemics (Non-HLP) after kidney transplantation

**Figure 3:**
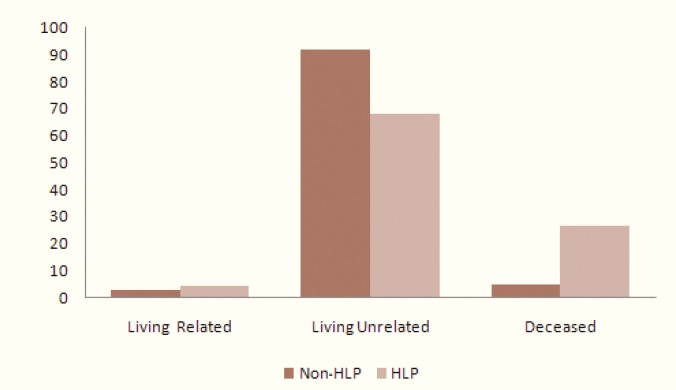
distribution of type of donation in hyperlipidemics (HLP) and non-hyperlipidemics (Non-HLP) after kidney transplantation

Only 12.7% (n=9) of all patients developed clinical IHD after successful kidney transplantation—five were hyperlipidemics. Eighty-nine percent were found with premorbidity (*i.e.*, hypertension, DM, or IHD), 22% had previous acute rejection episodes, 11% developed CMV infection, 45% were non-hypertensive, 55% were hypertensive, all were on dialysis before transplantation, and all received transplants from living unrelated donors. All hyperlipidemic patients who were taking azathioprine as premedicationfor a while, in spite of dietary modifications and use of antihyperlipidemics, were either hypercholestrolemic or hypertriglyceridemic for at least 24 months, while most of those who were not using the drug were normolipidemics within 6-24 months of transplantation.

The highest risk for graft deterioration in the hyperlipidemic group was 82% when it was associated with hypertension, while it was 18% in normotensive patients (p<0.005); when hyperlipidemia was associated with history of CMV infection, the risk was 68%, significantly (p<0.005) higher than that in non-deteriorated patients (32%). Presence of IHD was not associated with graft loss (p>0.05) (Fig 2).

## DISCUSSION

Although innovation of immunosupressive agents has improved the outcome of graft survival, association of these medications with various complications has caused challenges for patients and transplantation teams. Nowadays, the main bulk of chronic kidney diseases are systemic diseases that have serious chronic impacts on different vital organs (*e.g.*, hypertensive diseases and DM). In this study, our focus was mainly on the graft survival as a main vascular unit that was originally donated by obviously healthy human, so every graft deterioration would either be attributed to a *de novo* systemic disease process or to an immunogenic or sequalae of post-transplantation immunosupression therapy. The trigger for IHD is usually hyperlipidemia which causes atherogenesis leading to coronary stenosis. This sequence of vascular pathology can present itself in arterial and arterioral renal vascular system similar to that happens in the coronary arteries; nonetheless, the process is slow and silent leading to gradual renal graft deterioration. Currently, coronary artery disease takes the main bulk of adults morbidity and mortality among different world communities. Therefore, no matter if we treat atherogenesis as either an age-related phenomenon or secondary to post-transplantation phenomenon, more attention should be paid to the effect of atherogenesis on graft (a presumably healthy organ) than on coronary arteries to obtain a realistic view regarding the actual artherogenic effect of the immunosuppressive drugs. Fortunatly, only 2.7% of our patients developed IHD after successful kideny transplantion and 89% had premorbid conditions (*i.e.*, hypertension, IHD, and DM). The negligible rate of 2.7% enables us to look at graft function as a main indicator to analyze our findings. Kasiske, *et al*, claimed that 60%–80% of kidney transplanted patients developed hyperlipidemia within one year after transplantation [8]. Coresh, *et al*, found that 70%–80% of kidney transplanted patients developed hypercholestrolemia in immediate post-transplantation period [23]. We found that only 50% of patients developed post-kidney transplantation hyperlipidemia, 16% of whom had pre-transplantation hyperlipidemia. This wide variation in the reported incidence of hyperlipidemia is based on the fact that the main bulk of our sample was transplanted after the era of mycophenolate mofetile, thereby, our analysis of all hyperlipidemic patients who were receiving azathioprine as part of their immunosupression protocol, had extended a higher lipid profile level than those on mycophenolates. On the other hand, the dietary habits of Iranian community, which includes more fibers, make the lipid control easier. The insurance program of the Ministry of Health of Islamic Republic of Iran, for the transplanted patients, encouraged the patients and transplantation teams to apply fit follow up programs that resulted in early proper control of hyperlipidemia in these patients and minimized the atherogenic effects on the vascular system. The lower incidence of graft deterioration among hyperlipidemic patients (13%) in comparison to the non-hyperlipidemics (22%), directed the issue towards other associated factors that may have a more important role in graft function deterioration than hyperlipidemia. Bumgardner, *et al* (1995) and Hillbrand, *et al* (1999), did not find any association between graft function and hyperlipidemia. However, we found no significant association between hyperlipidemia and IHD in our patients yet, and clinical IHD is absent in non-hyperlipidemic transplanted group. Although, we had an excellent lipid control, we still had a high incidence of CMV infection and diseases among deteriorated hyperlipidemic paients (68%) in comparison to the rate of 32% in hyperlipidemics with non-deteriorated graft. Hypertensive diseases were observed in 82% of hyperlipidemic deteriorated patients as compared to 18% of hyperlipidemic non-hypertensives group. This association was similar in IHD after kidney transplantation, 68% CMV and 82% hypertension. The lower incidence of graft deterioration among patients with deceased donation (27%) or previous acute rejection episodes (40%) [18] uncovered the fact that modern immunosuppression is very effective in preventing and controlling of allogenic graft rejections, but none judged use of immunosuppression yield another mode of challenges like over immunosupression and undesirable side effects of immunosuppressive medications. However, the presence of post-transplantation hyperlipidemia without simultaneous clinical signs of IHD, made this association questionable.

## CONCLUSION

It seems that allograft rejection has a minor challenge in modern solid organ transplantation. The adverse effects of modern immunosuppressants have the main impact on longterm graft function. Post kidney transplantation hyperlipidemia is an associated biochemical phenomenon secondary to the use of immunosuppressive regimens, and has no obvious role in cardiovascular atherogenesis. The association between post kidney transplantation hyperlipidemia and hypertension or CMV infection makes the graft deterioration more likely.
